# The Saskatchewan/New Brunswick Healthy Start-Départ Santé intervention: implementation cost estimates of a physical activity and healthy eating intervention in early learning centers

**DOI:** 10.1186/s12913-017-1978-9

**Published:** 2017-01-19

**Authors:** Nazmi Sari, Nazeem Muhajarine, Amanda Froehlich Chow

**Affiliations:** 1 Department of Economics, University of Saskatchewan, Arts 815, 9 Campus Drive, Saskatoon, SK S7N 5A5 Canada; 2 Community Health & Epidemiology and Saskatchewan Population Health and Evaluation Research Unit University of Saskatchewan, Saskatoon, SK S7N 5A5 Canada; 3 College of Kinesiology, University of Saskatchewan, Saskatoon, SK S7N 5A5 Canada

**Keywords:** Physical activity, Implementation cost, Economic evaluation, Social return on investment, Early learning centres, Healthy Start-Départ Santé

## Abstract

**Background:**

Participation in daily physical activity and consuming a balanced diet high in fruits and vegetables and low in processed foods are behaviours associated with positive health outcomes during all stages of life. Previous literature suggests that the earlier these behaviours are established the greater the health benefits. As such, early learning settings have been shown to provide an effective avenue for exploring and influencing the physical activity and healthy eating behaviours of children before school entry. However, in addition to improving individual level health of children, such interventions may also result in a number of social benefits for the society. In fact, research among adult populations has shown that sufficient participation in physical activity can significantly lower hospital stays and physician visits, in turn leading to positive economic outcomes. To our knowledge there is very limited literature about economic evaluations of interventions implemented in early learning centers to increase physical activity and healthy eating behaviours among children. The primary purpose of this paper is to identify inputs and costs needed to implement a physical activity and healthy eating intervention (Healthy Start-Départ Santé (HS-DS)) in early learning centres throughout Saskatchewan and New Brunswick over the course of three years. In doing so, implementation cost is estimated to complete the first phase of a social return on investment analysis of this intervention.

**Methods:**

In order to carry out this evaluation the first step was to identify the inputs and costs needed to implement the intervention, along with the corresponding outputs. With stakeholder interviews and using existing database, we estimated the implementation cost by measuring, valuing and monetizing each individual input.

**Results:**

Our results show that the total annual cost of implementing HS-DS was $378,753 in the first year, this total cost decreased slightly in the second year ($356,861) and again in the third year ($312,179). On average, the total annual cost is about $350,000 which implies an annual cost of $285 per child. Among all inputs, time–cost accounted for the larger share of total resources need to implement the intervention. Overall, administration and support services accounted for the largest portion of the total implementation cost each year: 74% (year 1), 79% (year 2), and 75% (year 3).

**Conclusions:**

The results from this study shed lights for future implementation of similar interventions in this context. It also helps to assess the cost effectiveness of future interventions.

**Electronic supplementary material:**

The online version of this article (doi:10.1186/s12913-017-1978-9) contains supplementary material, which is available to authorized users.

## Background

It is well known that physical activity and healthy eating provide a number of health benefits for children of all ages [1]. Researchers suggest that the early years (0–5 years) is a critical period to establish physical activity and healthy eating patterns, as this stage of life lays the foundation for development of lifelong healthy living patterns [1–3]. Despite the benefits of engaging in these healthy behaviours, current research indicates that Canadian early years children spend a large portion of their day in sedentary behaviours and thus are engaging in lower than recommended daily physical activity; moreover their diets are often high in processed convenience foods containing excess fat and sugar [4–8]. These unhealthy behaviours have been associated with increases in overweight and obesity during the early years. Rates of overweight and obesity continue to rise among Canadian children, including those in their early years [9]. In North America, by school entry significant numbers of children 2 to 5 years old are already at risk for overweight or obesity [10]. Children who are overweight during the early years have an increased risk of being overweight or obese in later childhood and are four times more likely to become obese during adulthood [11, 12]. This evidence suggests the early years is the optimal time for establishing lifestyle patterns which incorporate healthy behaviours, that will in turn support children to achieve healthy weights over the course of their lifetime [13, 14]. Moreover, research has shown that developing the knowledge and skills to engage in health promoting behaviours at an early age can improve an individual’s health status later in life.

Compared to those reporting poor health status, individuals who are able to engage in behaviours which support their health and wellness, are more productive in the workforce, in turn reducing burden placed on the healthcare system and the economy [15, 16]. For instance, it has been shown that participation in physical activity increases wages and educational outcome of the individuals[17–20]. A body of literature also examined the impact of physical activity on physical and mental health as well as cognitive functioning [21–23]. These studies suggest that participation in health promoting behaviours has positive impact on cognitive functioning, health and well-being of the participants. While these positive effects suggest that physical activity generates private benefits to the individuals, there are a number of social benefits for the society. An obvious benefit in the context of publicly funded Canadian healthcare system is the likelihood of lower utilization of healthcare services as a result of participation in physical activity. This social benefit of physical activity has been shown in a large body of literature suggesting that participation in physical activity reduces length of stays at hospitals and use of physician services [24–33].

Given the number of individual and broader social benefits of engaging in healthy behaviours it is important to establish healthy lifestyle patterns at an early age. In order to establish such patterns children must be offered opportunities to engage in physical activity and healthy eating behaviours. Parents and early childhood educators (ECEs) are key actors in providing such opportunities for children in early years. As well, research shows that multiple factors in the social and physical environments where children live and play, interact and influence parental and educator abilities to provide physical activity and healthy eating opportunities [13]. Although parents and the home environment have an important influence on the development of children’s lifestyle patterns [34], it is important to note that over 54% of Canadian children ages six months to 4 years attend out of home care [35]. In addition to parents and the home environment, ECEs and early learning settings are therefore another major influence on children’s physical activity and healthy eating behaviours [34, 36]. Early learning settings can provide an effective avenue for exploring and influencing the physical activity and healthy eating behaviours of children and their educators. Experts suggest that educators and early learning centre environments can strongly influence children’s physical activity and dietary patterns [37–40]. Accordingly, centres have been identified as a promising setting for the delivery of interventions aimed at increasing the physical activity and healthy eating behaviours of children [2, 3, 41–44]. Early learning environments not only facilitate access to a large number of early years children, but also provide an ideal opportunity to introduce lessons, activities, and programming that reinforce physical activity and healthy eating [45].

As stated in recent systematic reviews, there are interventions implemented in early learning settings; however, these studies typically focus on either nutrition or physical activity [46, 47]. Although the short and long term benefits of combining physical activity and healthy eating for all ages are consistently shown in the literature, there are limited studies reporting the effectiveness and sustainability of interventions aimed at increasing both physical activity and healthy eating among preschool children in early learning settings. Larson et al., [48] found that only a small number of interventions reported in the existing literature were effective in improving both physical activity and healthy eating among children in early learning settings. As discussed above, some studies have also highlighted the economic benefits of engaging in healthy behaviours during adulthood. However, we are not aware of any economic evaluation of interventions conducted for this population group or this environment.

Healthy Start-Départ Santé (HS-DS) is one such intervention developed to promote opportunities for early years children to engage in and establish healthy behaviours (physical activity and health eating) while in early learning settings [49]. The multi-pronged intervention was implemented in early learning settings across Saskatchewan (SK) and in New Brunswick (NB). Early learning educators were trained to implement the HS-DS intervention in their early learning settings for 8 months. The intervention focused on incorporating opportunities for children to engage in and learn about physical activity and healthy eating.

In order to carry out a comprehensive economic analysis of this multi-pronged physical activity and healthy eating intervention and address gaps in the literature the primary purpose of this study is to identify inputs and costs needed to implement the HS-DS, and to conduct the first phase of a social return on investment (SROI) analysis for the HS-DS intervention. With this current work, we will focus on estimating the implementation cost of the intervention that will be used for a comprehensive economic evaluation that aims to estimate the social return on investment for the HS-DS intervention.

## Methods

### Healthy Start-Départ Santé Intervention, and the Program Logic Model

Developed and pilot-tested, HS-DS is a multi-pronged, inclusive, intersectoral and evidence-based intervention with the primary goal of promoting physical activity and healthy eating among Anglophone and Francophone children 3-5 years of age attending early learning centres (ELC) in SK and NB. The intervention’s characteristics are highlighted below; a comprehensive description of the HS-DS intervention is described elsewhere by Belanger and colleagues [49]. The vision is to ensure young children eat healthy and be physically active every day in order to achieve healthy weights. The mission is to encourage and enable families and educators to integrate physical activity and healthy eating in the daily lives of young children. The specific goals of the HS-DS intervention are as follows: 1) work with ECEs to implement HS-DS in English and French ELCs in urban and rural communities; 2) facilitate the engagement of partners; and 3) promote the uptake and implementation of key findings from the evaluation of the intervention through a knowledge development and exchange plan.

Underpinned by an adapted version of the healthy weights ecological model [50, 51] a number of interlinked components constituted the implementation of the HS-DS intervention in childcare centres. These six components are:

- the HS-DS Implementation Guide which is a resource designed to assist educators in implementing the intervention in their early learning centres. It is a step-by-step guide that walks educators through each stage of the intervention and it contains resources and ideas for increasing physical activities and health eating within the childcare centre;
- customized training, role modelling and monitoring of HS-DS in early learning centres. ECEs are trained to deliver the HS-DS intervention and midway through the implementation HS-DS trainers offer booster training to each centre. These trainings are customized to meet the unique needs of each centre. Throughout the intervention trainers offer the ECEs additional resources and support as needed.
- an evidence-based resource, LEAP-GRANDIR [52] which includes materials to increase physical activity and healthy eating in early learning settings. Specifically, Health Opportunities for Preschoolers (HOP) is a resource for children 3 to 5 years of age that focuses primarily on physical activity, but also includes physical literacy and healthy eating activities. In addition, educators receive Food Flair, a resource that contains healthy recipes and focuses on ways to create healthy eating environments. Each early learning centre also received a Healthy Start Active Play Equipment (APE) Kit, containing a number of supplies necessary for carrying out the activities described in the HOP binder;
- supplementary resources were offered to centres as needed. For example, some centres requested additional materials for increasing physical activity, indoors or during the winter months. As such, resources were provided as requested to address their specific needs;
- a knowledge development and exchange (KDE), and communication strategy with specific targets, messaging and material aimed to raise awareness and provide hands-on material for participants and community organizations; and
- engage in community partnerships to facilitate the implementation of the HS-DS intervention in early learning settings.

The intervention was designed to provide a step-by-step implementation using the implementation manual, while also including enough flexibility to adapt to local realities and incorporate additional resources as needed. The specific implementation steps involved first connecting with and building interest among ELC directors and educators to ensure ownership of the intervention. Second the intervention implementation within the ELCs involved training educators at the ELCs to use the implementation manual and demonstrating, hands-on, the multiple ways LEAP-GRANDIR resources (HOP and Food Flair) could be used in their early learning centre. During the training, educators received the necessary equipment/materials to implement Healthy Start in their centres (HOP and Food Flair, Healthy Start Active Play Equipment Kit and the Implementation Manual). The training was offered on site (in the ELCs) in one of three ways: a 3-h evening training, two 2-h evening trainings (4 h total), or one 5-h training during a weekend day. Centres could choose the format that worked best for them, and trainers adapted accordingly. Third, booster training sessions were held at the centres in the fourth and eighth month (if necessary). Fourth, providing ongoing support to educators as well as monitoring adherence and quality. Fifth, holding a meeting (in person or over the phone) with centre directors to discuss next steps and ensure sustainability.

**Fig. 1:**
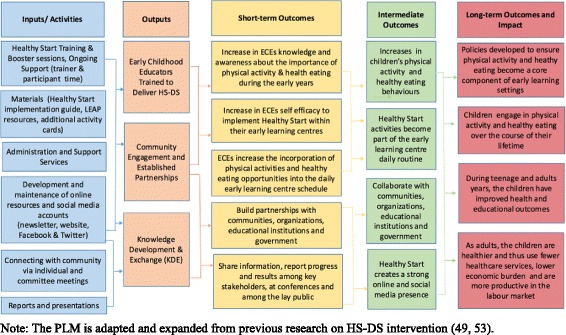
HS-DS Implementation Program Logic Model

The implementation of the HS-DS intervention took place over 8 months and incorporated a number of inputs that were directly linked to three intermediate outputs. A detailed Program Logic Model (PLM) has been created to depict the HS-DS intervention (see Fig. 1). The PLM depicted below has been adapted and expanded from previous research describing the pilot testing of the HS-DS intervention [49, 53]. Combined these inputs and corresponding outputs were associated with a number of short-term, intermediate and long-term outcomes. The short-term outcomes as shown in the PLM refer to increase in ECE’s knowledge and positive attitudes about the importance of physical activity and healthy eating during the early years and improvements in educator’s self-efficacy to deliver the HS-DS intervention and engage in activities with the children. Additionally, part of HS-DS’s short-term goals were to develop partnerships with community organizations and governments. Moreover, HS-DS aimed to provide ongoing feedback and engage partners through conference presentations and online venues. Intermediate outcomes identified within the PLM were that ELCs incorporated HS-DS into the daily routine within their centres and increased physical activity levels and healthy eating behaviours in children were observed. HS-DS also aimed to establish a strong online presence and share the intervention progress with partners, stakeholders and colleagues. Through these partnerships HS-DS also aimed to engage in community collaborations as an effort to increase the sustainability of HS-DS. Lastly, the long-term outcomes and potential impact of HS-DS stated that policies are created to ensure children in early learning settings are given opportunities to establish healthy behaviours at a young age. As they grow and mature they would have the knowledge, skills and confidence to continue to participate in physical activity and healthy eating throughout their life. In turn, children would have improved health and educational outcomes throughout their childhood and adult years, potentially decreasing their need for healthcare services and increasing their productivity in the labor market.

### Inputs and corresponding outputs for the HS-DS intervention

The first output was *ECEs Trained to Deliver HS-DS* in order to produce this particular output a number of inputs were required. These inputs included the *initial HS-DS training session.* As mentioned above the training was typically offered in the ELCs. Centres choose the format that worked best for them, and trainers adapted accordingly (3-h evening training, two 2-h evening trainings, or one 5-h).

Another type of training offered was a train-the-trainer model where a number of individuals interested in becoming HS-DS regional trainers were brought together and participated in a 2-day workshop. HS-DS trainers would travel to a community and hold the workshop over two days. Following this 2-day training the trainees would be certified HS-DS trainers and thus able to deliver HS-DS training to ELCs in their communities.

The second input was the *HS-DS Implementation Guide*, which is a step-by-step guide for implementing the intervention and thus promoting healthy eating and physical activity among early years children in care settings. This resource also provides educators with tools that can be used in early learning environments to assess their current environment and to offer ideas of how to incorporate more physical activity and healthy eating into children’s daily routines. All early learning environments that receive HS-DS training receive an implementation guide.

The third input was the *LEAP* resources/materials which was given to all centres receiving the intervention. LEAP is a set of evidence based resources that promotes healthy child development. ELCs trained in HS-DS receive HOP, an illustrated manual, containing child-tested physical activities for promoting active play among early years children. Along with the HOP manual, centres are given an APE kit containing a number of supplies necessary for carrying out the activities described in the HOP manual. ELCs that receive HS-DS are also given Food Flair, a healthy eating manual containing numerous ideas, activities and quality recipes that encourage and expose children to a variety of foods and food-related experiences. The total cost for HS-DS implementation materials given at the initial training including a snack provided during the training is $223.18 and this includes the LEAP resources, APE kit and Implementation guide.

Following the initial training, centres receive a *Booster Training Session* which are held at the centres in the fourth and eighth month (if necessary). Booster sessions range from 30 min to 135 min. They vary depending on the centre’s needs. In some centres they may be used to give new educators a mini HS-DS training. In other centres specific topics will be addressed as per requests of the centre.

The fourth input involved ongoing support and communication provided by HS-DS coordinators to centres during the intervention implementation period. During the 8-month intervention early learning centres were provided ongoing support which also allows for monitoring adherence and quality of the intervention delivery. The ongoing support was delivered through telephone and email communication. Centres were provided additional supports and resources as needed, such as photocopied physical activities focusing on particular skills. Centres are also able to access resources online from the *Healthy Start-Départ Santé Website*. In addition, centres received the bi-monthly *Healthy Start Newsletter* via email*.* During the final month of the intervention the HS-DS staff held a meeting (in person or over the phone) with centre directors to discuss next steps and ensure sustainability of HS-DS in the early learning centre program.

The second output was *Community Engagement and Established Partnerships* that require the primary input of *interacting and collaborating with community members*. During the intervention implementation the HS-DS management and implementation team made a conscious effort to meet with as many stakeholders in the communities where the project was rolling out. The HS-DS steering committee met three times per year to receive progress updates about the implementation and discuss steps for the expansion of HS-DS. The project was configured to move forward with a staggered regional large-scale roll-out that coincided with randomly selected centres in these same regions for the HS-DS evaluation. Each year the team expanded their community engagement activities throughout Saskatchewan via individual and committee implementation meetings. HS-DS met with many Early Years community organizations across Saskatchewan, Saskatchewan polytechnic and the Early Years Branch within the Government of Saskatchewan Ministry of Education.

The third intermediate output was *Knowledge Development and Exchange (KDE).* This output was addressed through the development of *online resources, social media, production of reports and presentations*. The web tools include the development of the *Healthy Start Website*, including the sharing of web articles. The production of the bi-monthly *Healthy Start Newsletter* was sent out to ELCs via email. In addition, the HS-DS team actively engaged in social media via Facebook and Twitter. In addition, HS-DS annual reports were produced for key stakeholders and the steering committee. Recently a comprehensive process evaluation report has been created to measure if the intervention was implemented as designed, participant satisfaction and level of adoption of the HS-DS intervention among ELCs.

Administration and support staff facilitated the delivery of all three intermediate intervention outputs. Specifically, the HS-DS implementation team was involved in providing the HS-DS training and ongoing support to participating centres. These staff members also facilitated community engagement and carried out implementation meetings with individual stakeholders. The KDE staff developed and updated the HS-DS website and the social media accounts (Facebook and Twitter). In addition, the staff created online newsletters and resources for those participating in the HS-DS intervention. The project manager was involved with over-seeing all components of the implementation. This included organizing three steering committee meetings per year and an annual implementation committee meeting. Together the project manager and KDE staff also composed an annual report for key stakeholders and steering committee members. Finally, the manager of the sponsoring organization provided accounting services and office space and materials (this included office space and storage, and use of an office telephone and photocopier).

### Analytical framework to estimate the implementation cost of the HS-DS

Following the relevant range of inputs identified in the previous section, each individual input was measured and valued. To do this, we started by measuring the quantities of each of the resources identified in the HS-DS database and through semi-structured interviews with HS-DS staff. The database, which was created in consultation with the HS-DS staff, contained information for each childcare centre about the trainings and booster sessions, including name, location and size of community in which the centre was located. There was also information regarding the length of the training and follow up booster sessions, number of trainers conducting the training, the trainers home base (regional or out of town) and how many educators and childcare staff attended the trainings and booster sessions. In addition to the inputs reported in the HS-DS database we conducted semi-structured interviews with the HS-DS project lead and manager. The interviews were guided by questions which were developed to gather detailed information about specific inputs associated with each output of HS-DS. During the interview the HS-DS staff were asked about administration, KDE and training activities along with their corresponding costs.

Next step in costing, we assigned unit cost or prices to monetize the inputs identified in quantities above. Among all inputs, time–cost accounted for the larger share of the total resources needed for the HS-DS intervention. It included time devoted (during training and booster sessions) by the ECEs, center directors, other childcare staff and trainers. In addition, as part of operational and administrative cost of HS-DS, and day-to-day business of the HS-DS intervention, there was time devoted by the HS-DS administration and support services staff. Their specific job titles and corresponding implementation duties are described in the previous section.

In order to monetize time–cost, we estimated the opportunity cost of trainees’ time during the training and booster sessions using their before-tax market wage rate. Given that we did not have exact wage rate for each trainee, we used an approximate wage rate using affiliations and job titles of the trainees. For Saskatchewan, we used average hourly wage rates for frontline ECEs reported in 2013 Saskatchewan Wage Survey after adjusting these rates for Saskatchewan inflation rate of 1.5% in 2013, 2.4% in 2014 and 1.6% in 2015 [54]. Saskatchewan Wage Survey, however, did not provide wage rates for the directors, and other trainees. To obtain wage rate for the directors, we estimated their wage rate using the 2007 provincial child care wage and fee survey [55]*.* The provincial survey reports the wage rate for ECEs and the directors as in 2007. Using these wage rates, we computed the relative wage rate for directors compared to the ECEs. To estimate the wage rate in 2015 for the directors we inflated the wage rate of ECEs using this relative rate. For others with no affiliation reported in the database, appropriate provincial minimum wage rate was applied. For New Brunswick we used the corresponding wage rate for directors and ECEs as reported in the New Brunswick 2014-2015 Child Day Care Services Annual Statistical Report [56]. For time cost of HS-DS staff (trainers/coordinators, communication officers, project lead and project manager), we used their actual wage rate as obtained from the HS-DS.

The hourly wages reported at the Saskatchewan Wage Survey and the New Brunswick 2014-2015 Child Day Care Services Annual Statistical Report were gross wages that did not include any benefits or other contributions paid by the employer. In order to compute the relevant cost to the employer, we added benefits and contributions paid by the employer to the gross wages. These additional components of cost are employment insurance (EI), contributions to the Canadian Pension Plan (CPP), the premiums for workers compensation, contributions to the supplementary pension plan (RRSP), and the premiums paid by the employer for health, vision and dental care.^1^

In addition to time–cost discussed above, there were other resources used in the intervention. Some are due to operational cost of the intervention (office space, and utilities for the HS-DS), others would be related to knowledge exchange activities (printing, newsletters, postage etc.,) or materials and manuals. The cost of these expendables was obtained from the HS-DS.

## Results

Findings from the cost analysis of HS-DS are described below. The total time commitment dedicated to training and booster sessions (for each province) is reported, followed by the monetization of these time-costs. All other inputs necessary for implementation of HS-DS are monetized and summarized by year to provide the overall cost of implementing HS-DS in Saskatchewan and New Brunswick.

### Time commitment for training and booster sessions

The time–cost of HS-DS training and booster sessions are summarized in the tables below. In order to create these tables, the first step was to extract and compile pertinent information from the HS-DS database regarding overall number of training or booster sessions delivered, the number of participants trained (educators, childcare centre directors and others), the number of trainers delivered the training, hours travelled by trainers and associated overnight stays. This information was presented by year and community size (where the childcare centres were located) in a supplementary file (see Additional file 1). To determine different types of communities, the Statistics Canada definition of Population Centre was used to categorize the communities as large (population of 100, 000 or more), medium (population of 30, 000-99, 000), small (population of 1000–29, 999) or rural (population of 999 or less)^2^ [57]. It is important to note that individual trainings and booster sessions with missing information were not included in these tables. In relation to data collected in Saskatchewan, one centre was removed from the training sessions and six centres were removed from the booster sessions due to missing data. All of the centres removed had received individual trainings rather than regional training. In New Brunswick no centres were removed due to incomplete or missing data, however only educators and centre directors participated in the HS-DS training. Moreover, information about the number of educators participating in the booster sessions was not recorded in New Brunswick. When comparing Saskatchewan and New Brunswick implementation activities, the HS-DS intervention was implemented in Saskatchewan for three years (2013/2014–2015/2016) and in New Brunswick for two years (2013/2014–2014/2015).

Using the data presented in the additional file, summary tables are created showing the required time commitment for an average training and booster sessions by province and community size. Tables 1 and 2 summarize the time commitment needed to implement the HS-DS intervention in SK and NB. The data for these tables was extracted from the 2015–2016 (SK) and 2014–2015 (NB) implementation training and booster session data since the dataset for this year was complete with no missing data (see Additional file 1).

**Table 1:** Average time dedicated for a training session in Saskatchewan & New Brunswick

	*Saskatchewan*				*New Brunswick*			
	Large	Medium	Small	Rural	Large	Medium	Small	Rural
*Participant’s time in training (hours)*								
Educators	56	15	47	31	3	9.6	7.1	7.5
Directors	4	3	4.8	4.4	3	3	3	3
Others	3.6	3	14	2.5	n/a	n/a	n/a	n/a
Trainers	5.5	6	6.6	6.3	6	6	6	6
*Time associated with travel for training*								
Travel time for trainers (hours)	0.9	4.8	5.6	2.4	0	4	4.1	3.2
Accommodation & per-diem (days)	0.1	0	0.5	0.3	0	0.4	0.9	1

*Note*: The table shows the average time dedicated for a training session by participants using data for 2015–2016 (SK) and 2014–2015 (NB). The bottom panel shows travel time and accommodation & per-diem for the trainers. All entries other than accommodation & per-diem are reported in hours. The average times (in hours and days) are an intermediate value that is applied to calculate costs for various activities in Table 3. Large communities have a population of 100 000 or more, medium communities have a population between 30, 000 and 99, 999, small communities have a population of between 1000 to 29, 999 and rural communities have a population of under 999

Source: our own computation

**Table 2:** Average time dedicated for a booster session in Saskatchewan & New Brunswick

	*Saskatchewan*				*New Brunswick*			
	Large	Medium	Small	Rural	Large	Medium	Small	Rural
*Participant’s time in training (hours)*								
Educators	7.6	0	4.1	4	0	0	0	0
Directors	1.3	0	1.4	1.4	0	1	1	0
Others	0.7	0	0.8	0.7	n/a	n/a	n/a	n/a
Trainers	1.3	0	1.4	1.7	0	1	1	0
*Time associated with travel for training*								
Travel time for trainers (hours)	0	0	5.1	6.1	0	3.3	2.7	0
Accommodation & per-diem (days)	0	0	0.3	0.3	0	1	0.8	0

*Note*: The table shows the average time dedicated for a training session by participants using data for 2015–2016 (SK) and 2014–2015 (NB). The bottom panel shows travel time and accommodation & per-diem for the trainers. All entries other than accommodation & per-diem are reported in hours. The average times (in hours and days) are an intermediate value that is applied to calculate costs for various activities in Table 3. Large communities have a population of 100 000 or more, medium communities have a population between 30, 000 and 99, 999, small communities have a population of between 1000 to 29, 999 and rural communities have a population of under 999

Source: our own computation

Table 1 summarizes the time dedicated to training and corresponding travel time needed to deliver the HS-DS training in SK and NB. The information in this table indicated that overall educator training hours were highest in large communities and lowest in medium sized communities (56 h and 15 h respectively) in SK; this was due to the fact that trainings in large communities tended to have the highest number of educator participation. However, the average number of training hours received by others (cooks and additional staff not including educators and directors) was highest in small communities and lowest in rural communities (14 h and 2.5 h respectively). The low average number of hours by others in centres located in rural communities was attributed to the fact that because staff from rural communities travelled to small communities to attend regional trainings, only a selected number of childcare staff (aside from educators) attended the training outside of their community. Trainer’s hours devoted to training were similar across centres in all community sizes, ranging from 6.6 h (large communities) to 5.5 h (small communities). Not surprisingly the average number of travel hours by trainers was highest for trainings in small communities (5.6 h) and lowest in large communities (0.9 h) in SK. This was due to the fact that trainers usually had to travel from large communities to small communities to deliver the trainings. Days for accommodation and per diem were most often recorded during trainings held in small communities. All remaining time commitments were similar across community sizes. It should be noted that in Saskatchewan few communities were categorized as medium sized, compared to communities of other sizes (large, small and rural). As a result, fewer trainings and booster sessions were held in medium sized communities.

The left panel of Table 2 summarizes the time dedicated to training and corresponding travel time needed to deliver an average HS-DS booster sessions in Saskatchewan. Educator booster training hours were highest in large communities (7.6 h) and lowest in medium communities where no trainings were held. In regards to average hours of training dedicated by trainers, the highest number of hours were reported in rural communities (1.7 h) and the fewest in medium sized communities, where no training hours were reported. Average travel hours were highest for trainers when delivering booster sessions in small and rural communities (5.1 and 5.6 h respectively). This was due to the fact that most booster sessions were delivered to centres individually and trainers had to travel from larger communities to deliver the booster trainings. Accommodation and per diem days reported by trainers travelling to small and rural communities were the same (0.3 days). No hours for travel or accommodation and per diem days were reported by trainers carrying out booster sessions in large and medium communities.

Tables 1 and 2 also present the average time in training and booster sessions and corresponding travel time needed to implement the HS-DS intervention in New Brunswick. The data used to create these tables were extracted from the 2014–2015 implementation database (Additional file 1: Table S2 and S4 presented in the Additional file 1) since the dataset for this year was complete. Specifically, the right panel of Table 1 shows the average time and other resources needed to deliver the HS-DS training in New Brunswick. Average educator hours devoted to training were highest in medium sized communities (9.6 h) and lowest in large communities (3 h). This is due to the fact that more trainings took place in centres located in medium sized communities.

As shown in Table 1, average training session hours for directors and trainers were the same across all community sizes in NB. Specifically, on average directors participated in 3 h of training and trainers delivered an average of 6 h of training. In relation to trainer’s travel time, average number of travel hours to small communities was the highest (4.1 h). Conversely, there were no hours of travel needed to deliver training in large communities. Trainer’s average travel time were similar for communities of all other sizes. On average, accommodation and per diem days during trainings were highest in rural communities (1 day) and lowest in large communities where no accommodation and per diem days were reported. This was attributed to the fact that trainers were travelling from the same large community to deliver the training in all other communities.

Table 2 also summarizes the average time and other resources needed to deliver HS-DS booster sessions in New Brunswick. There were no booster sessions held in centres located in large or rural communities and there was no record of educator hours for training in medium and small communities. Average training hours for directors and trainers were the same for medium and small communities (1 h). In relation to trainers’ hours of travel, the highest travel time were reported in medium sized communities (3.3 h), compared to small communities (2.7 h). Similarly, the highest number of accommodation and per diem days were associated with travel to medium sized communities (1 day), this time was marginally less in small communities (0.8 days). These findings were due to the fact that trainers were almost always required to travel to deliver booster trainings, as their home base was located in the same large community.

When comparing Saskatchewan and New Brunswick time commitments for carrying out HS-DS trainings, overall results indicated that in Saskatchewan training hours among educators were highest in large communities. Conversely, in New Brunswick educators training hours were highest among centres located in medium sized communities. Participation in training among directors varied by community size in Saskatchewan, with highest average number of hours reported in small communities (4.8 h). However, in New Brunswick the average number of hours in which directors participated in trainings was the same across all community sizes (3 h). In relation to trainers’ travel time, travel to trainings was highest in small communities for both Saskatchewan and New Brunswick. Overall, compared to Saskatchewan, New Brunswick trainers were more likely to report accommodation and per diem days during HS-DS trainings.

Average time–cost comparisons for carrying out HS-DS booster sessions in Saskatchewan and New Brunswick indicate some differences, however there were also some similarities observed. Comparisons could not be made between provinces for educator hours of training because no booster sessions were reported in centres located in large or rural communities in New Brunswick. Moreover, educator hours for booster training were not reported for in centres located in medium and small communities. In relation to booster training, the average number of hours’ directors participated in training sessions was similar for small communities in Saskatchewan (1.4 h) and New Brunswick (1 h). Average travel time for trainers in Saskatchewan was highest for trainings in small communities (5.5 h). However, travel time for trainers in New Brunswick was highest in medium sized communities (3.3 h). Overall, during booster session delivery New Brunswick reported more accommodation and per diem time than Saskatchewan (1 day and 0.3 days respectively). This was attributed to the fact that New Brunswick trainers were only based out of one community whereas Saskatchewan trainers were based out of different locations around the province, thus reducing trainer travel time in Saskatchewan.

### Cost of training and booster sessions

The monetary cost of carrying out HS-DS training and booster sessions is presented in Table 3. Specifically, the table compares the average cost of conducting a training or a booster session in Saskatchewan versus New Brunswick. These costs are reported in Canadian dollars and presented as cost per session, and cost per trainee for each community size (large, medium, small and rural). In order to calculate these costs, the time-costs per average session reported above (Tables 1 and 2) were monetized using corresponding wage rates (including benefits), travel allowance and daily per diem rates (respectively).

**Table 3:** Average cost of a training and a booster session in Saskatchewan & New Brunswick (in 2015 CAD$)

	*Saskatchewan*				*New Brunswick*			
	Large	Medium	Small	Rural	Large	Medium	Small	Rural
*Training session*								
Cost per session	1558	728	1717	1096	302	529	483	468
Cost per trainee	98	104	125	126	151	126	143	134
*Booster session*								
Cost per session	248	n/a	340	369	n/a	138	123	n/a
Cost per trainee	33	n/a	77	104	n/a	138	123	n/a

*Note*: All values in the table are shown in 2015 Canadian dollar using the most recent available data in SK (2015–16) and NB (2014–15). Large communities have a population of 100 000 or more, medium communities have a population between 30, 000 and 99, 999, small communities have a population of between 1000 to 29, 999 and rural communities have a population of under 999

Source: our own computation

The average cost of training in Saskatchewan compared to New Brunswick, appears to be higher per session; conversely, cost per trainee appears to be greater in New Brunswick. This is due to the fact that cost per session is based on the number of trainees in each training session. Thus, as the number of trainees participating in each training session increases (decreases), the corresponding cost per session will also increase (decrease). On average, compared to New Brunswick, Saskatchewan training sessions had higher participation rates (regardless of community size). As such, the overall cost per session for each community size was greater in Saskatchewan than in New Brunswick. However, the cost per trainee is more informative when assessing the cost efficiency of any training or booster session. When comparing the cost per trainee, results indicate that on average it costs more to train a participant in New Brunswick compared to Saskatchewan. This is due to two key factors: The first, trainers in New Brunswick were all based out of the same large community, however most of the trainings were held outside of this community. Thus, the trainers typically had to travel to deliver the intervention training. The second factor was that on average fewer trainees were trained at each session and the trainer to trainee ratio was lower in New Brunswick. For example, in some cases two trainers travelled to train one trainee. Thus compared to Saskatchewan, New Brunswick trainers often had to travel farther to deliver trainings for fewer trainees.

Within province comparisons (by community size) suggest that in Saskatchewan carrying out trainings in large communities was the most cost efficient ($98 per trainee), and the least cost efficient trainings took place in rural communities ($125 per trainee). This can be attributed in part to the fact that trainers often travel from other (small, medium or large) communities to rural communities to deliver HS-DS trainings. Moreover, the average number of trainees at a session tends to be greatest in large communities.

In New Brunswick trainings in medium sized communities tend to be least costly ($126 per trainee) compared to trainings in large communities ($151 per trainee). As discussed above this can also be attributed to the fact that more trainees tended to attend the trainings in medium communities. Thus the greater the trainer to trainee ratio the lower the cost per trainee for a training.

As presented in the lower panel of Table 3, the average cost per booster session was lower compared to the average cost per training session. This is attributed to the fact that on average booster sessions were approximately 60 min long, compared to training sessions which were at least 180 min long. As with training sessions, the cost per session was largely dependent on the number of trainees in a given session. Thus, when comparing cost per session to cost per trainee, the cost per trainee is (as stated above) more informative when assessing the cost efficiency of any training or booster session. Comparisons between provinces indicate that in Saskatchewan, compared to New Brunswick, the average cost per booster session was greater, however the average cost per trainee was lower. Results from within province comparisons suggest that in Saskatchewan carrying out booster sessions in large communities was the most cost efficient way to deliver the booster session. This was due to the fact that compared to booster sessions in medium, small and rural communities, more trainees attended booster trainings in large communities. Conversely, in New Brunswick the cost per session and cost per trainee was the same because the trainers were typically only training one or two educators in a booster session.

These results presented in Table 3 can be used in order to estimate the total training cost of a similar training elsewhere. For this purpose, one should use per trainee cost to estimate the total cost of training delivered in a comparable community. For instance, if a training would be replicated in a large community the way that it has been delivered in Saskatoon (a large community in Saskatchewan), then the expected cost would be about $100 for each individual trained. However, it will be 50% higher ($151 per trainee) if one would replicate a training using the training model of a large community in New Brunswick. For booster sessions, corresponding results presented in Table 3 should be used for the same purpose.

### Total cost of the HS-DS intervention in 2013–2016 period

The total annual cost for each year of implementing the HS-DS intervention in Saskatchewan and New Brunswick is presented in Table 4. The overall cost of the training and booster sessions for each year was calculated using the average cost per session (Table 3) and the total number of sessions held in each community size (see Additional file 1). As part of the initial training the trainers in Saskatchewan a train-the-trainer model was also employed and the cost of this model was added to the total training and booster session costs in Saskatchewan for year one. While the total cost for training and booster sessions was presented by province, all other costs of implementing the intervention (administration & support, materials, online services and social media, reports and other KDE activities and community engagement) were combined for Saskatchewan and New Brunswick.

**Table 4:** Total cost of HS-DS implementation (in 2015 CAD$)

	2013–2014	2014–2015	2015–2016
Training and booster sessions			
Saskatchewan	59,093	35,961	51,052
New Brunswick	4,405	8,638	N/A
Materials for training	9,150	8,035	6,026
Administration & support services			
Wages & benefits	260,588	262,105	214,214
Others	19,776	21,231	20,048
Online services & social media	6,000	2,000	2,000
Reports & other KDE activities	7,470	6,370	6,370
Community engagement and partnership	12,271	12,521	12,469
*TOTAL COST (2015 CAD$)*	*$378,753*	*$356,861*	*$312,179*

*Note*: Other administrative and support services include office supply and postage, office space and storage, telephone charges, and staff training

Source: our own computation

The HS-DS intervention involved additional activities that were not directly related to the implementation, such as an evaluation of the interventions and administrational and accounting duties required by the funding organization. Additionally, HS-DS was housed within and sponsored by an existing community organization. As such, the HS-DS staff and sponsor organization staff only devoted a portion of their time implementation activities. Therefore, the cost of administration & support services was calculated using only a portion of the wages paid to the HS-DS implementation staff, KDE staff, project manager and manager of the sponsor organization. The material costs are computed by multiplying the cost of all materials (such as the HS-DS implementation guide, LEAP resources and APE kits) given to centres during implementation by the number of centres that received the intervention each year. The annual use of online services and social media includes activation and/or maintenance for the HS-DS website, social media accounts (Facebook and Twitter) and *Mailchimp.*

Reports and other KDE activities included the design and postal delivery of three stakeholder reports that were sent to committee members each year. In addition, electronic newsletters, fact sheets and brochures were created and electronically distributed to the public. Other KDE materials include the cost of a photo shoot which was used to provide images for online materials and resources. In addition, promotional materials with HS-DS branding and logo (toques, cups, pedometers. APE kit I.D. tags and t-shirts) were manufactured and distributed to participating childcare centres as motivational resources and prizes.

The last component contributing to the overall cost of implementing the HS-DS intervention are the activities associated with community engagement and building partnerships. These activities included three steering committee meetings held each year with approximately 15 individuals from both middle and upper managerial positions. The meeting were 3 h long and focused on the ongoing progress of the HS-DS intervention. Implementation advisory meetings were also held with individual stakeholders and one HS-DS staff member on a monthly basis (12 meeting per year). Lastly, an annual implementation advisory committee meeting was held via conference call with the majority of the implementation committee (approximately 10 members). The meetings were 2 h in length and focused on the intervention progress and upcoming activities.

**Fig. 2:**
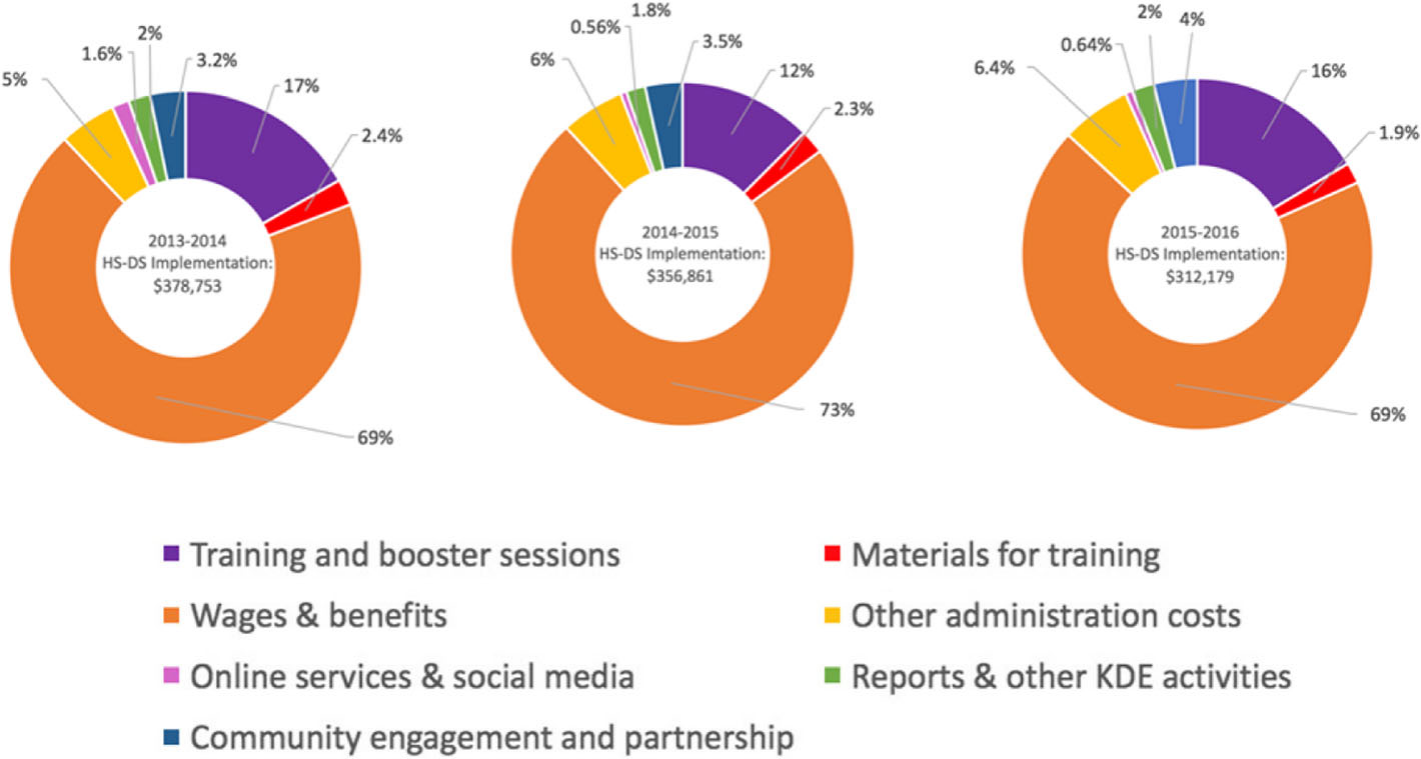
Percentage of total cost for each input component of HS-DS implementation in Saskatchewan & New Brunswick, 2013–2016

Figure 2 illustrates the percentage of total cost for each input component of HS-DS implementation for each year. Overall results in the figure indicated that the cost of trainings and booster sessions accounted for approximately 17% (year 1), 12% (year 2) and 16% (year 3), of yearly expenditures of the implementation of the HS-DS. Given that New Brunswick held fewer trainings and only conducted trainings over a 2-year period, the cost of training and booster sessions in New Brunswick only accounted for approximately 1% and 2% of the training and booster session costs during year 1 and 2 (respectively). Administration and support services (which included staff wages, office supplies and postage, office space and storage, telephone charges, and staff training.) accounted for the largest portion of the total implementation cost each year: 74% (year 1), 79% (year 2), and 75% (year 3). Less costly were community engagement and partnership activities which accounted for approximately 3–4% of the total annual implementation cost. The remaining inputs (materials, online services & social media, and reports & other KDE activities) each contributed to under 3% of the annual cost of implementing HS-DS. The cost for material and reports and other KDE activities was similar to each year. Conversely, cost of online services and social media decreased from the first year (accounting for 1.6% of the cost) to less than 1% of the total cost in the second and third years.

The total cost of implementing HS-DS slightly decreased each year from $378,753 (year 1), $356,861 (year 2) and $312,179 (year 3). This can be attributed in part to the fact that certain expenses were a one-time cost paid in the first year. Additionally, New Brunswick did not implement HS-DS in any centres during the third year.

## Discussion

The results of this study indicated that the overall cost of implementing the intervention decreased each year. This was attributed to two factors: first, a number of initial expenses were incurred at the onset of the intervention and thus were a one-time cost paid during the first year; and the second factor was that the intervention as not implemented in New Brunswick during the third year. Each year the largest annual expenditures were associated with administration and support services, particularly the wages and benefits paid to HS-DS staff involved with the intervention implementation. These costs were by far the largest expense accounting for at least three quarters of the annual cost each year. The second largest annual cost was related to the training and booster session delivery. Given that New Brunswick implemented HS-DS on a smaller scale and did not carry out HS-DS trainings during the third year, the majority of the training and booster session took place in Saskatchewan.

Our results showed that delivering the HS-DS training and booster sessions were less cost efficient in New Brunswick compared to Saskatchewan. This was attributed to the fact that trainers in New Brunswick typically had to travel significant distances to deliver the trainings. Additionally, on average, fewer trainees were trained at each session and the trainer-to-trainee ratio was lower in New Brunswick. Additionally, although the initial expense of the train-the-trainer model applied in Saskatchewan increased the overall training cost during the first year, this approach reduced the travel costs associated with delivering the training and booster sessions over the 3-year implementation period. This was due to the fact that train-the-trainer trainees resided in various communities across Saskatchewan and thus they were able to carrying out a number of the trainings delivered to educators in nearby communities, thus reducing travel time. While one needs to take the specific circumstances into account when replicating such an intervention, it is also essential to explore cost efficient ways to deliver the training relevant to the context. In this sense, our results provide a guideline for cost of a potential replication of this intervention.

In particular, as this paper provides estimates on costs of implementing the intervention in two Canadian provinces, it informs the policy makers in terms of potential financial commitment required in other provinces contemplating similar interventions. Our cost estimates would also be relevant for international audiences with similar contextual circumstances to the two provinces, for example, spread of population settlements, distance to cities and towns, and population density. Even when cost estimates reported here are not directly comparable to other contexts, our costing methodology would still be helpful to any jurisdiction contemplating physical activity and healthy diet interventions in pre-school age populations, as it provides a detailed guide to identify inputs and estimate corresponding costs in a variety of contexts.

As with any research, there are limitations identified within this study; particularly related to limited data about exact time-costs dedicated to some implementation activities. Thus, in some cases (as indicated) assumptions were made based on existing information and memory recall of HS-DS staff. In addition, we used estimated wages rather than actual wages to assess and monetize the time devoted to training activities. Additional sensitivity analysis and robustness check are needed to evaluate the reliability of our estimates, and they should be incorporated to the next phase of the SROI analysis.

## Conclusion

The purpose of this paper was to conduct the first phase of a SROI analysis of HS-DS, a multi-pronged physical activity and healthy eating intervention implemented in ELCs across Saskatchewan and New Brunswick. Specifically, this phase of the analysis focused on estimating the annual implementation cost of the intervention over the course of three years (2013–2106). Given the limited SROI research focusing on health promoting interventions implemented in ELCs, this study contributes to a gap in the current literature.

As discussed earlier, the total annual cost of implementing HS-DS is about $350,000 ranging from $312,179 (year 3) to $378,753 (year 1). In a given year during the course of the intervention, there were more than 1000 children in 50 to 60 ELCs that were influenced by the intervention. In other words, the intervention, on average, had an impact on 1230 children ranging from 1052 children (year 1) to 1444 children (year 3). These numbers and the estimated annual cost of the intervention suggest that the annual cost per child would be around $285 ranging from $216 (year 3) to $360 (year 1). Given that these results are only the first step in a comprehensive economic evaluation of the HS-DS intervention, the readers should not make any inference regarding the potential social return from this intervention. While these findings help us to understand the size of the intervention cost, further research conducting the next phase of the SROI analysis is needed to examine and assess the rate of social return from this intervention.

## Additional file

Additional file 1: Table S1. Training sessions by year and community size (large, medium, small and rural) in Saskatchewan. **Table S2.** Training sessions by year and community size (large, medium, small and rural) in New Brunswick. **Table S3.** Booster Sessions by year and community size (large, medium, small and rural) in Saskatchewan.**Table S4.** Booster Sessions by year and community size (large, medium, small and rural) in New Brunswick. (PDF 271 kb)

## Abbreviations

APE: Healthy Start Active Play Equipment
CAD: Canadian dollars
CPP: Canadian Pension Plan
ECEs: Early childhood educators
EI: Employment Insurance
ELCs: Early learning centres
HOP: Health Opportunities for Preschoolers
HS-DS: Healthy Start-Départ Santé
KDE: Knowledge development and exchange
n/a: Not applicable
NB: New Brunswick
PLM: Program Logic Model
RRSP: Registered Retirement Saving Plan
SK: Saskatchewan
SROI: Social return on investment

## Footnotes

1 The employer contribution shares for the EI and CPP are 2.632% and 4.95% respectively. For the EI the maximum insurable earning is $50,800 [58]. For the CPP there is an exemption of $3,500. After that the CPP is paid up to a maximum income of $53,600 [59]. Workers compensation premium is 1.10% determined by the Saskatchewan Workers Compensation Board for the industry group S21 [60]. The minimum annual premium is $100 and maximum assessable wage is $69,242 [60]. The RRSP contribution by the employer is assumed to be 6% and other health benefits (dental and vision, prescription drugs) are assumed to be a fixed amount of $2500 per year.

2 A population centre has a population of at least 1,000 and a population density of 400 persons or more per square kilometre, based on the current census population count. All areas outside population centres are classified as rural (999 or less). Population centres are classified into three groups, depending on the size of their population: small population centres, with a population between 1,000 and 29,999; medium population centres, with a population between 30,000 and 99,999; and large urban population centres, with a population of 100,000 or more.
