# Supplementary material for: The Saskatchewan/New Brunswick Healthy Start-Départ Santé intervention: implementation cost estimates of a physical activity and healthy eating intervention in early learning centers
Source: BMC Health Serv Res. 2017 Jan 19;17:57. doi: 10.1186/s12913-017-1978-9 (PMC5247800; doi:10.1186/s12913-017-1978-9)
Supplement: Additional file 1: Table S1. — Training sessions by year and community size (large, medium, small and rural) in Saskatchewan. Table S2. Training sessions by year and community size (large, medium, small and rural) in New Brunswick. Table S3. Booster Sessions by year and community size (large, medium, small and rural) in Saskatchewan.Table S4. Booster Sessions by year and community size (large, medium, small and rural) in New Brunswick. (PDF 271 kb) [file 12913_2017_1978_MOESM1_ESM.pdf]

Additional file 1

The Saskatchewan/New Brunswick Healthy Start-Départ Santé Intervention: Implementation cost estimates of a physical activity and healthy eating intervention in early learning centers[1]

Nazmi Sari[*]
Department of Economics, University of Saskatchewan

Nazeem Muhajarine
Community Health & Epidemiology and Saskatchewan Population Health and Evaluation Research Unit, University of Saskatchewan

Amanda Froehlich Chow
College of Kinesiology, University of Saskatchewan

[1] This project was funded by the Public Health Agency of Canada (PHAC). During the course of the project we have received assistance from the HS-DS managerial staff from Saskatchewan and New Brunswick. We thank the PHAC for financial support, and the HS-DS for their assistance. While data set for this work, in part, was provided by the HS-DS, the analysis and conclusions drawn are the responsibility of the authors.

[*] Correspondence: Nazmi Sari, Department of Economics, University of Saskatchewan, Arts 815, 9 Campus Drive, Saskatoon, SK, Canada S7N 5A5; E-mail: Nazmi.Sari@usask.ca; homepage.usask.ca/~sari/

Table S1: Training sessions by year and community size (large, medium, small and rural) in Saskatchewan

	2013-2014				2014-2015				2015-2016			
	Large	Medium	Small	Rural	Large	Medium	Small	Rural	Large	Medium	Small	Rural
Total # of sessions	15	4	9	5	5	3	8	4	11	1	8	7
Total # of hours	45	11	27	15	21	11	32	14	44	3	38	31
Total # of educators trained	109	40	73	22	64	42	59	13	153	5	79	50
Total # of directors trained	15	4	9	5	5	3	8	4	11	1	8	7
Total # of others trained	14	7	20	5	8	6	9	1	10	1	23	4
Total # of trainers					6	5	9	5	15	2	11	10
Total # of hours travelled by trainers	0	11.2	37.1	15	5	11.8	24	28.3	9.9	4.8	44.8	17.1
Total # of overnight stays by trainers	0	0	0	0	1	2	3	2	1	0	4	3

Note: Large communities have a population of 100 000 or more, medium communities have a population between 30, 000 and 99, 999, small communities have a population of between 1000 to 29, 999 and rural communities have a population of under 999.

Table S2 Training sessions by year and community size (large, medium, small and rural) in New Brunswick

	2013-2014				2014-2015			
	Large	Medium	Small	Rural	Large	Medium	Small	Rural
Total # of sessions	1	2	5	0	1	5	8	2
Total # of hours	3	6	15	0	3	15	24	6
Total # of educators trained	6	11	32	0	1	16	19	5
Total # of directors trained	1	2	5	0	1	5	8	2
Total # of trainers	2	4	10	0	2	10	16	4
Total # of hours travelled by trainers	0	6.1	19.6	0	0	20	33	6.3
Total # of overnight stays by trainers	0	2	4	0	0	2	7	2

Note: Large communities have a population of 100 000 or more, medium communities have a population between 30, 000 and 99, 999, small communities have a population of between 1000 to 29, 999 and rural communities have a population of under 999.

Table S3: Booster Sessions by year and community size (large, medium, small and rural) in Saskatchewan

	2013-2014				2014-2015				2015-2016			
	Large	Medium	Small	Rural	Large	Medium	Small	Rural	Large	Medium	Small	Rural
Total # of sessions	5	1	5	1	7	5	9	4	7	0	10	18
Total # of hours	2.75	1	6.75	1.5	8.25	6	12	5	9.42	0	14.08	31
Total # of educators trained	41	6	35	7	37	20	47	10	41	0	29	42
Total # of directors trained	5	1	5	1	6	5	8	4	7	0	9	15
Total # of others trained	3	1	5	1	1	2	5	1	4	0	6	7
Total # of trainers	5	1	5	1	8	5	9	6	7	0	10	18
Total # of hours travelled by trainers	.83	1.73	11.27	1.73	15	22	49.37	38.9	0	0	50.87	110.33
Total # of overnight stays by trainers	0	0	0	0	1	2	1	1	0	0	3	6

Note: Large communities have a population of 100 000 or more, medium communities have a population between 30, 000 and 99, 999, small communities have a population of between 1000 to 29, 999 and rural communities have a population of under 999.

Table S4 Booster Sessions by year and community size (large, medium, small and rural) in New Brunswick

	2013-2014				2014-2015			
	Large	Medium	Small	Rural	Large	Medium	Small	Rural
Total # of sessions	0	1	4	0	0	2	5	0
Total # of hours	0	1	4	0	0	2	5	0
Total # of educators trained	0			0	0			0
Total # of directors trained	0	1	4	0	0	2	5	0
Total # of trainers	0	1	4	0	0	2	5	0
Total # of hours travelled by trainers	0	3.07	16.53	0	0	6.6	13.7	0
Total # of overnight stays by trainers	0	1	3	0	0	2	4	0

Note: Large communities have a population of 100 000 or more, medium communities have a population between 30, 000 and 99, 999, small communities have a population of between 1000 to 29, 999 and rural communities have a population of under 999.
